# Response to “Practice what you preach: Ensuring scientific spheres integrate Indigenous Peoples’ and Local Communities’ rights and agency too” by Lopez-Maldonado

**DOI:** 10.1007/s13280-021-01676-x

**Published:** 2021-12-02

**Authors:** Victoria Reyes-García, Álvaro Fernández-Llamazares, Yildiz Aumeeruddy-Thomas, Petra Benyei, Rainer W. Bussmann, David García-del-Amo, Natalia Hanazaki, Ana C. Luz, Pamela McElwee, Vicky J. Meretsky, Zsolt Molnár, Isabel Ruiz-Mallén, Matthieu Salpeteur, Eduardo S. Brondizio

**Affiliations:** 1 Institució Catalana de Recerca i Estudis Avançats (ICREA), Barcelona, Spain; 2 Institut de Ciència i Tecnologia Ambientals, Universitat Autònoma de Barcelona, Universitat Autònoma de Barcelona 08193 – Bellaterra, Barcelona, Spain; 3 Faculty of Biological and Environmental Sciences, Helsinki Institute of Sustainability Science (HELSUS), University of Helsinki, Helsinki, Finland; 4 Functional and Evolutionary Ecology, University Montpellier, CNRS, Montpellier, France; 5 Department of Ethnobotany, Institute of Botany and Bakuriani Alpine Botanical Garden, Ilia State University, Tbilisi, Georgia; 6 Departamento de Ecologia e Zoologia, Universidade Federal de Santa Catarina, Florianópolis, Brazil; 7 ISEG- Lisbon School of Economics & Management, Universidade de Lisboa, Lisbon, Portugal; 8 Department of Human Ecology, Rutgers University, New Brunswick, NJ USA; 9 O’Neill School of Public and Environmental Affairs, Indiana University, Bloomington, IN USA; 10 MTA Centre for Ecological Research, Hungarian Academy of Sciences, Vácrátót, Hungary; 11 Internet Interdisciplinary Institute, Universitat Oberta de Catalunya, Barcelona, Spain; 12 Patrimoines Locaux, Environnement et Globalisation (PALOC), French National Research Institute for Sustainable Development (IRD), Muséum National d’Histoire Naturelle, Paris, France; 13 Department of Anthropology, Indiana University Bloomington, Bloomington, USA

Comment to: Lopez-Maldonado, Y. 2021. Practice what you preach: Ensuring scientific spheres integrate Indigenous Peoples’ and Local Communities’ right and agency to. *Ambio*. https://doi.org/10.1007/s13280-021-01663-2

In our work (Reyes-García et al. 2021), we primarily address the current moment of international negotiations of the new post-2020 biodiversity framework that will directly affect Indigenous Peoples and local communities (IPLC). We argue that current biodiversity negotiations and ensuing policies should be grounded on respecting IPLC rights and agency, including the recognition of Indigenous and local knowledge (ILK) in environmental governance. The paper aims to reaffirm that IPLC hold knowledge essential for governance of biodiversity; that IPLC conceptualizations of nature can help sustain local livelihoods and influence global visions; that IPLC engagement in biodiversity policy contributes to the recognition of human rights; and that IPLC inclusion is essential to ensure they can exercise their rights to territories and resources. Points raised by Lopez-Maldonado in her comment (2021) to our piece are broadly relevant to research related to ILK systems, but our work does not focus on this issue.

Western science certainly has often been a colonial enterprise (McAlvay et al. 2021). However, there have also been engagements of respect and voluntary co-production between scientists and IPLC. Different IPLC have chosen to engage with non-local and non-Indigenous scientists and researchers and to work together around common agendas (e.g., Fernández-Llamazares et al. 2021), for which it is not productive to critique all science and all scientists without acknowledging these alternative relations. Indeed, there are currently many efforts to reframe power relations among knowledge systems, and our work is part of these reframing efforts.

Our paper results from an assessment done within the framework of the Intergovernmental Science-Policy Platform on Biodiversity and Ecosystems Services (IPBES), which has developed one of the most inclusive processes to bring together several sources of knowledge to tackle a global challenge: biodiversity loss. From its conceptual framework, IPBES has promoted dialogue across different knowledge systems (Díaz et al. 2015). Moreover, beyond a deliberate framework that facilitates recognition of different knowledge systems from the start, IPBES has also mobilized funding and engaged networks of stakeholders with diverse worldviews (McElwee et al. 2020). The approach followed in IPBES includes procedures for assessments of nature and people’s linkages with nature (including IPLC), participatory mechanisms, and institutional arrangements for IPLC inclusion throughout the process (Hill et al. 2020). Indeed, this pioneering approach makes important steps in supporting ILK systems by respecting rights, strengthening communities and their knowledge systems, and supporting knowledge exchange through dialogue (Hill et al. 2020). While much can be improved, the IPBES approach attempts to move beyond the colonial, extractive, or dismissive efforts of those who do not value different knowledge systems. The approach also proposes new pathways for an inclusive and participatory mechanism, aligning with propositions made by IPLC themselves.

We acknowledge—as we did in our original piece- that we are non-Indigenous scholars with scientific backgrounds and that this is a privileged positionality. We also acknowledge that our best attempts to reframe relations between different knowledge systems are insufficient to undo historical injustices. We wish they were. But we also note that the academic community can help to signal that biodiversity negotiations and ensuing policies should be grounded on respecting IPLC knowledge, rights and agency, particularly in a context in which they are not. Not recognizing the value of this contribution can become counterproductive both in promoting dialogue and in finding new ways to address global challenges. More solidarity is required for transformative change to occur.
